# RSK2 activity mediates glioblastoma invasiveness and is a potential target for new therapeutics

**DOI:** 10.18632/oncotarget.13084

**Published:** 2016-11-04

**Authors:** Florian J. Sulzmaier, Shirley Young-Robbins, Pengfei Jiang, Dirk Geerts, Amanda M. Prechtl, Michelle L. Matter, Santosh Kesari, Joe W. Ramos

**Affiliations:** ^1^ Cancer Biology Program, University of Hawaii Cancer Center, University of Hawaii at Mānoa, Honolulu, HI 96813, USA; ^2^ Department of Translational Neuro-Oncology and Neuro-therapeutics, John Wayne Cancer Institute at Providence Saint John's Health Center, Santa Monica, CA 90404, USA; ^3^ Department of Pediatric Oncology, Erasmus University Medical Center, Rotterdam, 3015 GE, The Netherlands

**Keywords:** glioblastoma, GBM, invasion, ERK, RSK, MAPK

## Abstract

In glioblastoma (GBM), infiltration of primary tumor cells into the normal tissue and dispersal throughout the brain is a central challenge to successful treatment that remains unmet. Indeed, patients respond poorly to the current therapies of tumor resection followed by chemotherapy with radiotherapy and have only a 16-month median survival. It is therefore imperative to develop novel therapies. RSK2 is a kinase that regulates proliferation and adhesion and can promote metastasis. We demonstrate that active RSK2 regulates GBM cell adhesion and is essential for cell motility and invasion of patient-derived GBM neurospheres. RSK2 control of adhesion and migration is mediated in part by its effects on integrin-Filamin A complexes. Importantly, inhibition of RSK2 by either RSK inhibitors or shRNA silencing impairs invasion and combining RSK2 inhibitors with temozolomide improves efficacy *in vitro*. In agreement with the *in vitro* data, using public datasets, we find that RSK2 is significantly upregulated *in vivo* in human GBM patient tumors, and that high RSK2 expression significantly correlates with advanced tumor stage and poor patient survival. Together, our data provide strong evidence that RSK inhibitors could enhance the effectiveness of existing GBM treatment, and support RSK2 targeting as a promising approach for novel GBM therapy.

## INTRODUCTION

Glioblastoma (GBM) is an aggressive cancer that arises from glial cells and their progenitors and is often diagnosed at a late stage [1, 2]. GBM cells are highly motile and invade the healthy brain tissue surrounding the primary tumor site. This makes a complete surgical resection of GBM difficult, contributing to high recurrence and mortality rates in GBM patients [3, 4]. Furthermore, invading GBM cells are less sensitive to the current standard therapies including radiation and chemotherapy with DNA alkylating agents such as temozolomide [5, 6]. GBM cells spread within the brain by migrating in a mesenchymal fashion along the extracellular matrix (ECM) of myelinated fiber tracts and blood vessels [7, 8]. This migration is mediated by dynamic interactions between the cancer cell and its surrounding ECM through transmembrane signaling receptors including the integrins [9, 10].

The MAPK/ERK (Mitogen-activated protein kinase/extracellular signal-regulated kinase) pathway reciprocally regulates integrin signaling [11, 12]. MAPK/ERK signaling controls integrin activity in part through the downstream target 90 kDa ribosomal S6 kinase 2 (RSK2) [13, 14]. RSKs are serine/threonine kinases that activate transcription and proliferation, and regulate apoptosis [15–17]. RSK inhibitors have revealed additional roles for RSKs in controlling proliferation of prostate and breast cancer cells [18–20] and differentiation of muscle cells [21]. Recent work has shown that RSKs are required for invasion and metastasis of many cancers [22–24]. All RSK isoforms contain two kinase domains, a central regulatory linker domain, and a carboxyl-terminal ERK docking site [16, 25, 26]. The N-terminal kinase domain (NTKD) is in the AGC kinase family and appears to be responsible for phosphorylation of all substrates. The C-terminal kinase domain (CTKD) is in the CaM kinase family and regulates the NTKD. Activation of RSKs requires phosphorylation at multiple sites. Initially ERK phosphorylates the CTKD at Thr573 and a linker site at Ser369. The CTKD then phosphorylates Ser380 and thereby generates a docking site for PDK1. PDK1 binds and phosphorylates Ser221 in the NTKD [27, 28]. Phosphorylation at Tyr529 by either FGF receptor 3 (in myeloid cells) or Src and Fyn (in fibroblasts) can enhance inactive ERK binding to RSK2 [29, 30]. RSK2 directly phosphorylates filamin A (FLNa). FLNa is a large protein that forms an elongated homodimer that crosslinks F-actin and affects invasion [31–33]. Activation of RSK2 by ERK promotes FLNa phosphorylation and enhances its binding to integrin complexes thereby modulating focal adhesion composition and activity [34]. FLNa binds to the integrin cytoplasmic tail at an NPxY sequence that also interacts with talin [35]. As a result, FLNa and talin may compete for integrin-binding [35]. Finally, FLNa association with the integrin cytoplasmic tails maintains the integrin in the inactive state [36].

GBMs harbor alterations in receptor tyrosine kinases such as MET and epidermal growth factor receptor (EGFR) and proteins downstream in the Ras-MAP kinase and the PI3 kinase-AKT-mTOR pathways [37]. Other important pathways in GBM are Wnt, sonic hedgehog, Rb, and p53 [38]. We here focus on the activation of the Ras-MAP kinase pathway, which in its turn activates RSKs. Deregulation of the Ras-ERK MAP kinase pathway contributes significantly to the development, progression, and invasiveness of GBM. An activating mutation in EGFR resulting from deletion of the extracellular variant III region occurs in 20-30% of all primary GBMs, while amplification of EGFR is seen in 40-70% of primary GBMs [37, 38]. In addition mutations or amplifications have also been identified in MET, and downstream of EGFR in Ras [39], B-Raf [39, 40] and RSK2 [41]. Finally, therapeutics that block invasion can also increase chemotherapeutic sensitivity of GBM [42].

RSK2 acts as mediator of cell migration and invasion, thereby driving tumor aggressiveness. Since GBM is characterized by a high degree of invasiveness, we hypothesized that RSK2 signaling is involved in the progression of this malignancy. In this study, we report that RSK enzymatic activity is an important regulator of GBM invasiveness *in vitro*. RSK co-localizes with FLNa at the plasma membrane and modulates GBM cellular adhesion. We find that targeting RSK's enzymatic activity results in reduced *in vitro* GBM cell motility and invasion. Moreover, combining perturbation of RSK function with standard chemotherapy temozolomide enhanced temozolomide's effectiveness in patient GBM cells resistant to temozolomide. In addition, we show that RSK2 is upregulated in human GBM patient cells, correlates with tumor grade, and is a significant predictor of poor patient survival. Our findings support targeting RSK enzymatic activity as a potential novel therapeutic approach for GBM.

## RESULTS

### RSK2 activity is required for GBM cell migration and invasion

**Figure 1:**
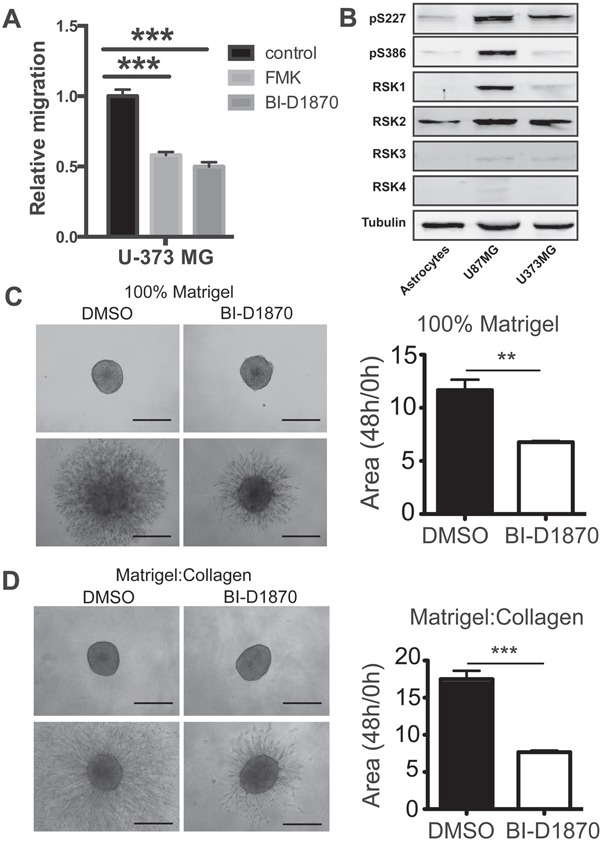
RSK isoforms are required for GBM migration and invasion **A.** Migration of U-373 cells was determined in the presence of RSK inhibitors (FMK and BID1870) or control DMSO. Relative migration into the scratch was measured at 24 hours. **B.** Immunoblot showing expression of RSK1-4 isoforms in the indicated cells. **C.** Day 4 U-373MG tumor spheroids were embedded in either 100% matrigel or **D.** a 50% Matrigel/50% collagen mixture (right panel) and treated with DMSO or 10 μM BI-D1870. Images were acquired at 0, 24, and 48 hours after addition of drug. Bar graphs show the quantification of the normalized area of the spheroids as the mean of 3 independent experiments (carried out in duplicates, n = 6).

We previously found that RSK2 kinase activity is the driving force behind its regulation of cellular motility [14]. Thus, we predicted that RSK2 activity is required for GBM cell migration. We therefore tested the effects of RSK inhibition on *in vitro* migration of an established GBM-derived cell line (U-373 MG). Treatment with the RSK inhibitors FMK and BI-D1870 impaired transwell cell migration along a fibronectin/EGF gradient in these cells (Figure 1A). All four RSK isoforms appear to be expressed in all GBM cell lines tested at levels higher than in the control astrocytes (Figure 1B). Loss of RSK activity also inhibited GBM cell invasion, as RSK inhibitor treatment of U-373 MG cells resulted in impaired three-dimensional outgrowth (Figure 1C, D).

**Figure 2:**
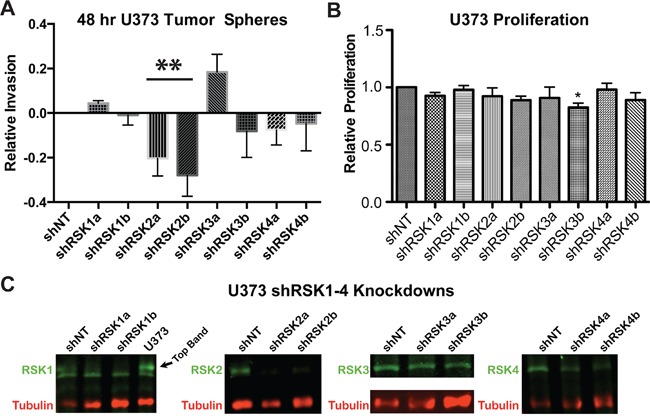
Individual RSK isoforms regulate GBM cell invasion in 3D **A.** Stable U-373 MG cell lines with knocked down RSK1, -2, -3, or -4 isoform expression (shRSK1-4) or cells carrying a scrambled control vector (scr) were generated using two independent shRNA constructs targeting RSK1-4. RSK1-4 knock-down and control cell lines were subjected to a tumor spheroid invasion assay. Spheroids were embedded in a 50% matrigel-50% collagen I matrix and invasion was analyzed after 48 hours. Quantification at 48 hours is shown. **B.** RSK1-4 isoform knock-down had no effect on GBM cell viability at 48 hours. **C.** Protein knock-down levels were determined by immunoblotting as indicated.

The RSK inhibitor BI-D1870 inhibits all four RSK isoforms while FMK inhibits RSK1, -2, and -4. We therefore determined if RSK2 specifically was required for invasion using shRNA silencing in U373 cells. We found that U373 cells were dependent on RSK2 for invasion (Figure 2A), Cell viability was not affected in these treatments (Figure 2B) and the level of knockdown of each RSK isoform is shown (Figure 2C). These findings confirm the requirement of RSK2 kinase activity for GBM tumor invasion.

### RSK2 co-localizes with FLNa and modulates GBM cellular adhesion

**Figure 3:**
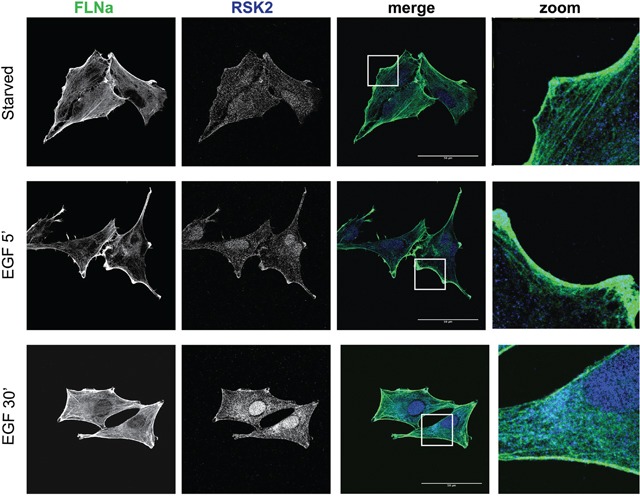
RSK2 co-localizes with FLNa U-373 MG cells were grown on coverslips coated with 10 μg/ml fibronectin. Cells were serum starved overnight and then stimulated for 5 or 30 min with 10 ng/ml EGF. Cells were fixed and stained for RSK2 and FLNa using specific primary antibodies. Immunostaining was visualized with confocal microscopy using a 63x objective. Scale bars shown represent 30 μm (5 μm in the zoom pictures).

Integrin-based cell adhesion is a crucial regulator of mesenchymal cancer cell migration [43]. We previously reported that RSK2 controls cell motility in HeLa and neuroblastoma cells in part by changing integrin activation status and hence adhesion due to FLNa phosphorylation and subsequent FLNa association with integrin tails [14, 44]. We therefore examined whether RSK2 co-localizes with FLNa in migrating U373MG cells. EGF stimulation increased the density of cortical actin in membrane ruffles. We found that in addition to nuclear translocation, we saw an association of RSK2 and FLNa at the cell membrane (Figure 3). Since RSK2 co-localizes with FLNa in migrating cells, it could influence migration through direct phosphorylation of FLNa as previously described [34].

**Figure 4:**
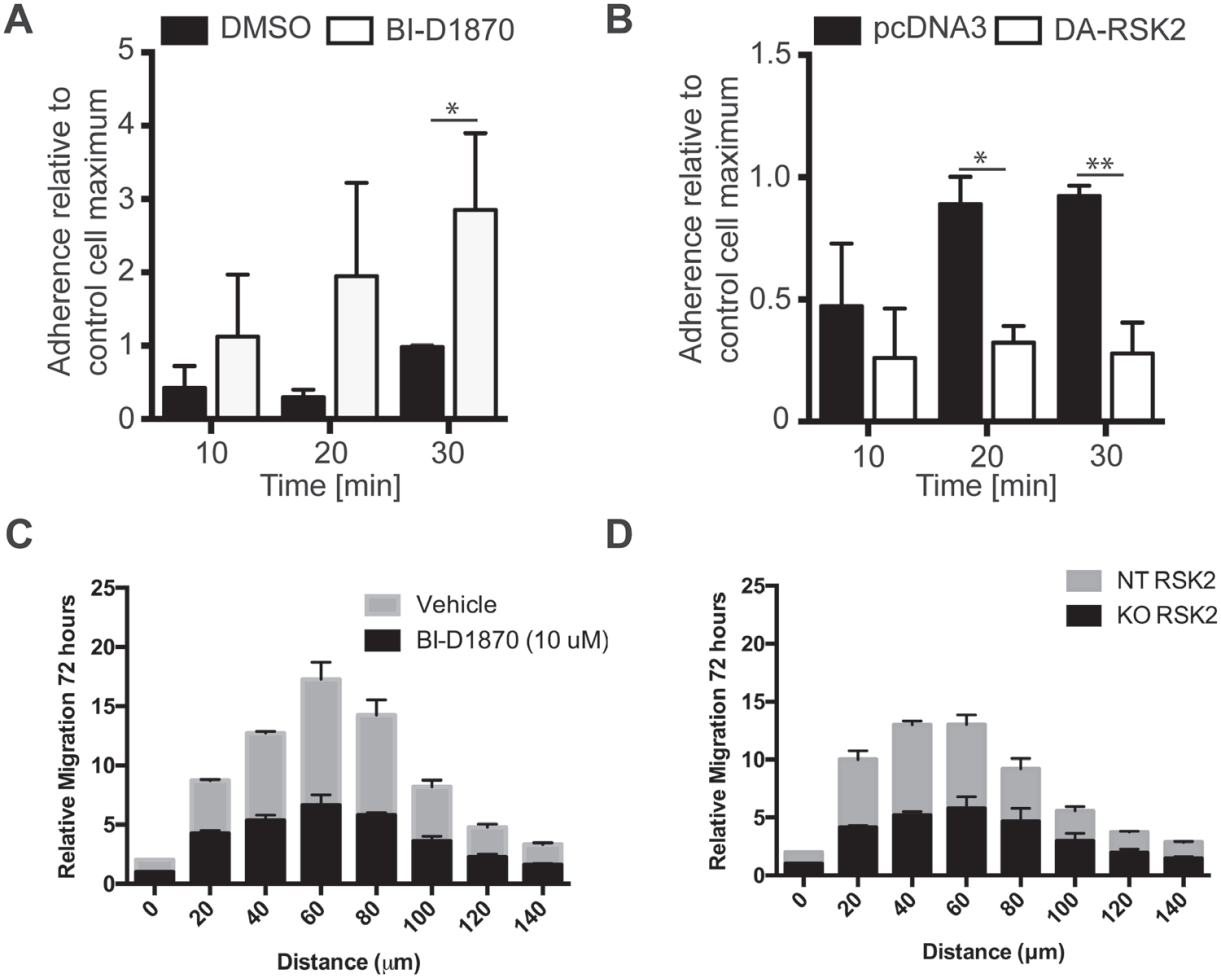
RSK2 activity reduces cell adhesion to fibronectin and invasion into brain slices We examined early adhesion of U-87 MG cells on culture plates coated with 3FN-(9–11). Before plating, cells were **A.** pre-treated with 10 μM BI-D1870 or DMSO carrier control or **B.** transiently transfected with DA-RSK2 or a pcDNA3 empty vector control. 48 hours after transfection or 15 min after pre-treatment cell adhesion was measured. Bar graphs show the number of cells adherent after the indicated amount of time. The experiment was carried out in 3 independent repeats. Error bars shown as SEM. Statistically significant differences are marked with asterisks (* P < 0.05, ** P < 0.01, *** P < 0.001). Scale bar: 500 μm. **C** and **D.** We examined the ability of U373 cells treated with BI-D1870 inhibitor (C) or with RSK2 knocked out using CRISPR/Cas9 (D). Whole brain slices of 300 μm thickness were placed on the membrane of a six-well plate culture insert. GBM cell lines U373MG, U373MG with RSK2 gene knock-down (KO-RSK2), or U373MG with a non-targeting shRNA (NT-RSK2) were labeled with the PKH67 fluorescent linker. After 7 days, one small spheroid of approximately 200 μm was transferred to each brain slice as close to the corpus callosum as possible. The co-cultures were maintained for an additional 72 hours. To quantify the invasiveness of the spheroids, the density of the fluorescent signal was measured in each 20 μm section using ImageJ Software. Shown are the migration depths. Error bars are shown as SEM.

We hypothesized that RSK2 also controls integrin activity in GBM cells, resulting in changes in cellular adhesion and migration. We therefore determined if RSK activity had direct impact on GBM adhesion. We found that cells treated with the RSK inhibitor BI-D1870 were significantly more adherent to 3FN-(9–11), the recombinant cell-binding domain of fibronectin (Figure 4A). Loss of RSK2 activity affected cell adhesion as early as 10 min after plating the cells. Conversely, transient transfection of a dominant active mutant of RSK2 (RSK-Y707A) impaired cell attachment, resulting in reduced overall adhesion at multiple time-points (Figure 4B).

GBM invasion in the brain requires association of the GBM cells with a complex microenvironment that is not fully replicated using 2D and 3D matrices. Furthermore, the current RSK inhibitors are quickly degraded *in vivo* and are therefore not suitable for testing *in situ* in mouse xenograft models. We therefore examined the effect of RSK inhibition with BI-D1870 or RSK knock-out using CRISPR/Cas9 deletion on U373 GBM cell invasion into mouse brain slices. These slices are 200 μm thick and are cultured in media to maintain an environment more relevant to *in vivo* invading GBM tumor cells. In both tests we found significantly reduced invasion when RSK activity is reduced (Figure 4C, 4D). Together these results support the conclusion that RSK2 activity is a potent regulator of integrin-dependent cell adhesion and GBM cell migration and invasion.

### RSK2 activity drives patient-derived GBM cell migration and invasion

**Figure 5:**
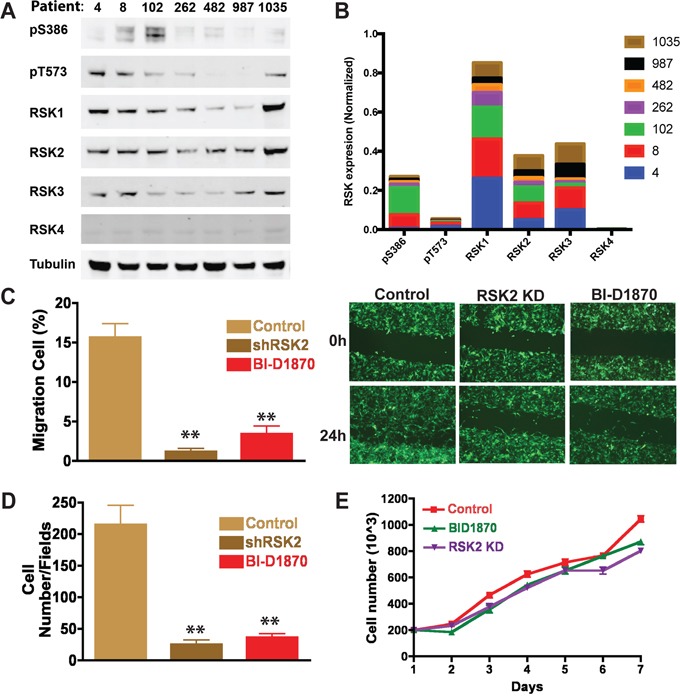
Patient-derived GBM cells require RSK2 for invasion and migration **A.** RSK1-4 are expressed in patient cells and are phosphorylated at S386 and T573 indicating likely activation. **B.** Quantification of RSKs normalized to tubulin with cumulative expression in patients indicated for each. **C.** Quantification of scratch assay migration experiments and **D.** transwell assay. At 24 hours, RSK2 gene knock-down cells and wild-type cells treated with BI-D1870 significantly decreased cell migration in both scratch (t-test; P = 0.0036 and 0.00043, respectively) and transwell assays (t-test; P = 0.0061 and 0.011, respectively). Images of example scratch assays are shown (C). Scratch assays were quantified using ImageJ and normalized to time 0 h. **E.** There was no effect on cell number or morphology in the migration assays.

Since GBM-derived cell lines, whether cultured on plastic or on brain slices, are not perfect representatives of primary GBM cells, we examined if RSKs were similarly required for migration and invasion of fresh GBM patient-derived cells. We found that RSKs are expressed in GBM cells from many different patients (Figure 5A, B). Activity of RSKs, as indirectly measured using phospho-epitope antibodies to pS386 and pT573, also was elevated in some patient cells (Figure 5A). We concluded that increased RSK2 levels may contribute to GBM progression and promote tumor recurrence. We therefore tested the effects of RSK2 gene knockout or chemical inhibition on the migratory capabilities of the patient-derived primary GBM cell line SK748. This cell line was uniquely maintained as suspended tumor neurospheres, an *in vitro* culture technique in which cell lines better retain characteristics consistent with fresh patient cells [45, 46]. Scratch migration assays with SK748 showed that both RSK2 knock-out and chemical inhibition (with BI-D1870) decreased cell migration (Figure 5C). Similarly, RSK2 gene knock-down with shRNA and chemical inhibition significantly blocked GBM SK748 invasion in transwell assays with epidermal growth factor (EGF) and fibroblast growth factor (FGF) as chemoattractants (Figure 5D). Neither RSK2 gene knock-down nor chemical inhibition affected cell viability, proliferation, or morphology in these assays, as confirmed through microscopic analysis (Figure 5E). RSK2 kinase activity is therefore required for primary patient-derived GBM migration.

### RSK inhibition enhances the effectiveness of standard GBM therapy

**Figure 6:**
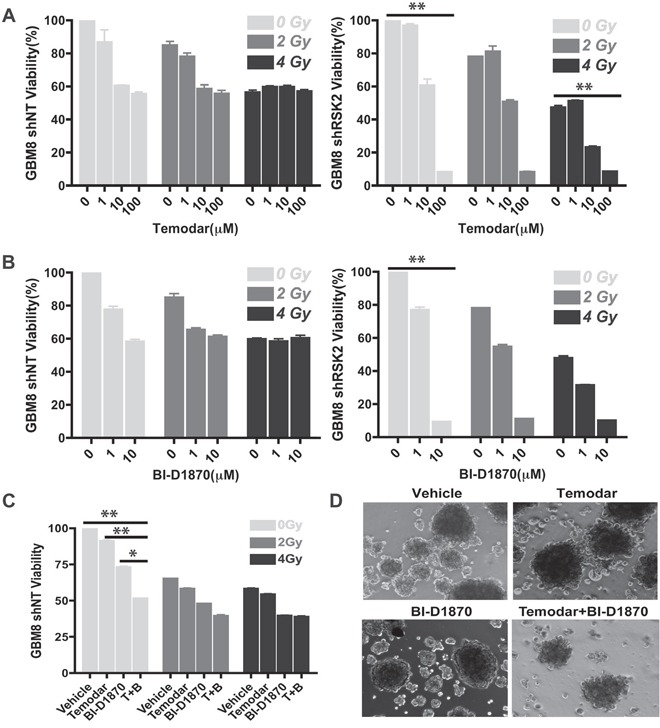
Inhibition of RSK2 sensitizes resistant patient-derived GBM to temozolomide We investigated the effect of RSK2 in combination with temozolomide treatment and irradiation on GBM patient cells that were resistant to chemotherapy and radiation. **A.** RSK2 gene knock-down sensitized GBM cells to temozolomide and the RSK1-4 inhibitor BI-D1870. At high temozolomide concentrations, the cell viability decreased from 57% to 8% (P < 0.001). In addition, 10 μm RSK inhibitor BI-D1870 decreased cell viability from 58% to 9% (P < 0.001). RSK2 gene knock-down had no significant effect on radiation response. **B.** RSK Inhibitor BI-D1870 enhanced the anti-tumor effect of temozolomide in RSK2 gene knock-down cells. For resistant wild-type cells, temozolomide/BI-D1870 combination treatment (both at 1 μM), decreased cell viability to 51% as compared to single reagent treatment effects of 91% and 72% remaining viability (P < 0.001 and P < 0.01, respectively). **C** and **D.** The combination of temozolomide and BI-D1870 was more effective at killing GBM cells than either alone.

GBM invasion targeting has been shown to enhance the effectiveness of the current standard therapies temozolomide and irradiation [3]. We therefore tested whether RSK2 function interference would enhance the effectiveness of temozolomide and irradiation therapy using GBM neurospheres derived from a patient with temozolomide resistance (GBM8) *in vitro*. We found that GBM8 cells were resistant to temozolomide and irradiation, with cell viabilities > 50% at 100 μM drug or 4 Gy irradiation treatment. Combined treatments did not increase cell death. Interestingly, RSK2 gene knock-down sensitized the patient cells to temozolomide, especially at higher concentrations (10 μM and 100 μM; Figure 6A). RSK2 gene knock-down also improved the anti-tumor effect of BI-D1870 as the cell viability dramatically decreased while we noted little change in control cells containing non-targeting control shRNA (Figure 6B). This is probably due to the sub-optimal effects of RSK2 shRNA function and/or compensation by additional RSK isoforms. Importantly, although low concentrations of either temozolomide or BI-D1870 alone had little effect on cell proliferation, combinations showed an additive anti-tumor effect (1 μM temozolomide with 1 μM BI-D1870; Figure 6C, 6D). Therefore, the combination of RSK inhibition with temozolomide is a promising new therapeutic approach in GBM treatment.

### RSK2 expression is elevated in human glioma tissue, correlates with high tumor grade, and is prognostic for poor patient outcome

**Figure 7:**
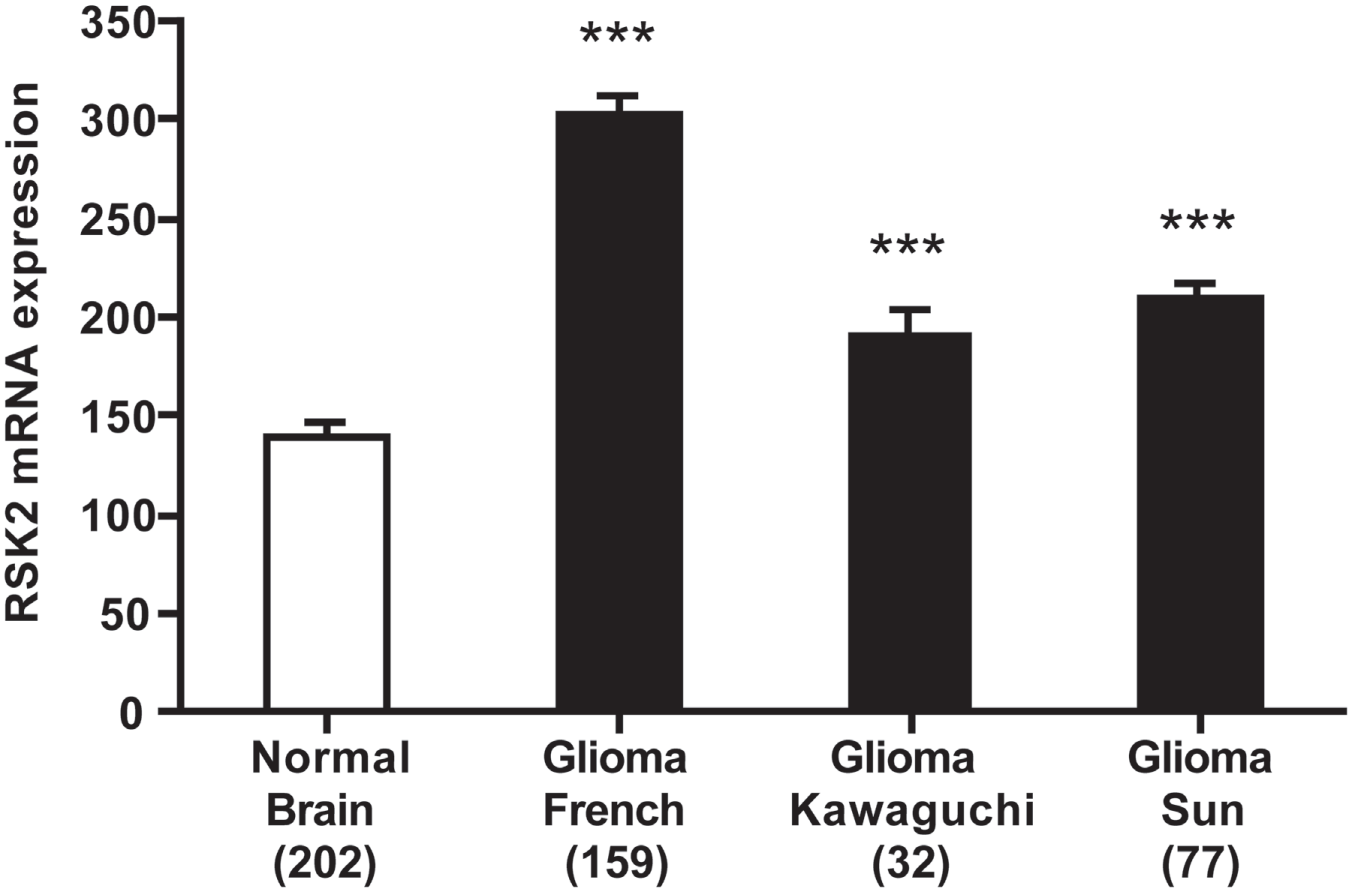
RSK2 mRNA expression is higher in human glioma than in normal brain tissue Differential RSK2 mRNA expression between the largest collection of normal human brain tissue (from the Roth-504 “Human Body Map”), and the three public human glioma datasets used in further analyses. RSK2 expression is significantly lower in normal brain than in glioma tissue, although glioma mRNA expression can vary between datasets. Y-axis presents RSK2 mRNA expression, X-axis dataset (in bracket the number of samples in the analysis). For presentation reasons, only GBM (stage 4) samples were used, but the differences remain significant when all dataset samples are analyzed (results not shown). Bars represent average ± SEM. * P < 0.05, ** P < 0.01, *** P < 0.001.

**Figure 8:**
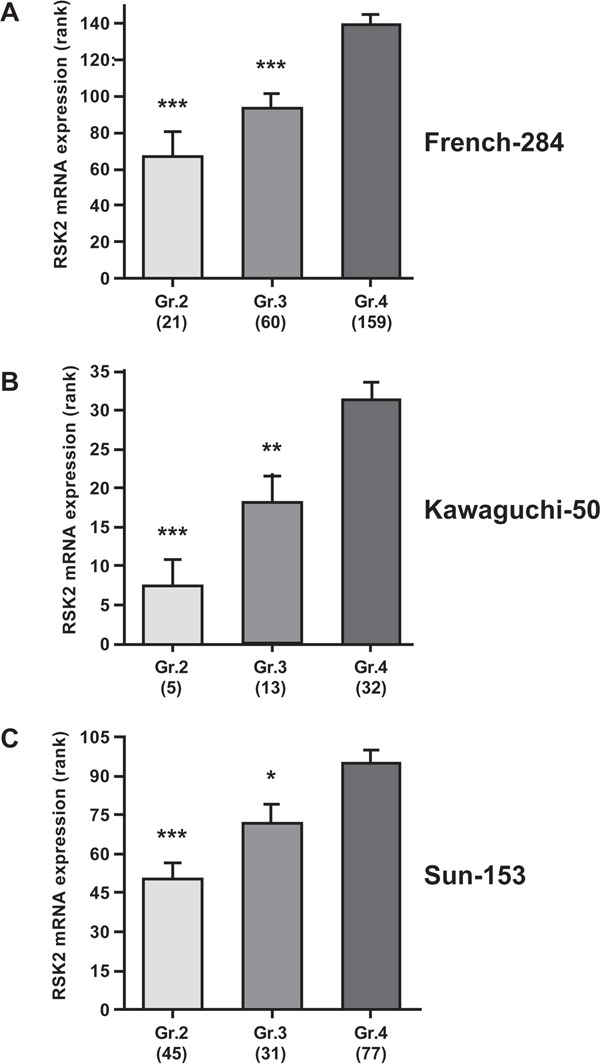
RSK2 mRNA expression correlates with high human glioma tumor stage Correlation of RSK2 tumor mRNA expression with WHO glioma tumor grade (grades 2-4) was analyzed using the Kruskal-Wallis test. Shown are graphs representing the three largest public glioma datasets with full WHO grade annotation: **A.** French-284, **B.** Kawaguchi-50, **C.** Sun-153). Y-axis presents RSK2 tumor mRNA expression (rank-based), X-axis WHO-grade. In all three datasets, RSK2 expression is significantly higher in stage 4 than in stage 2 or 3 tumors. Bars represent average ± SEM. * P < 0.05, ** P < 0.01, *** P < 0.001.

**Figure 9:**
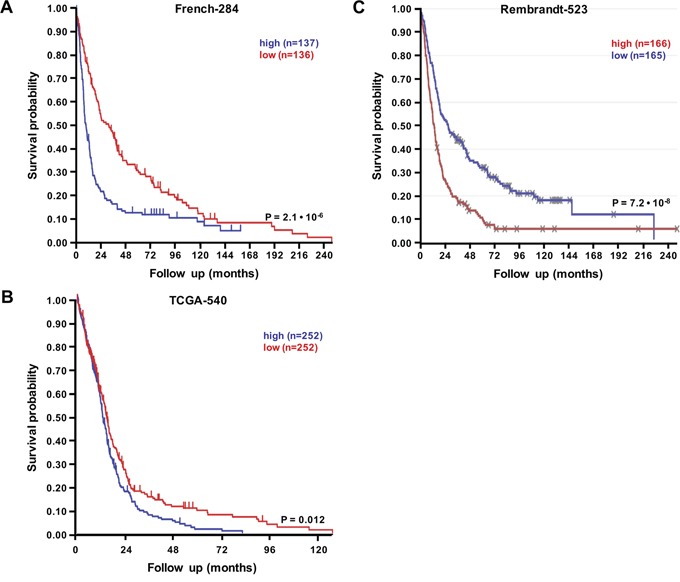
RSK2 mRNA expression is prognostic for poor glioma patient prognosis Prognostic significance of RSK2 tumor mRNA expression in human glioma patients as determined by Kaplan-Meier analysis. Shown are Kaplan-Meier graphs representing the overall survival prognosis of glioma patients based on high or low RSK2 tumor mRNA expression in the three largest public glioma datasets with survival data: **A.** French-284, **B.** TCGA-540 (both screenshots from the R2 website), and **C.** REMBRANDT-523 (screenshot from the Betastasis website). It should be noted that the color indications for patients with high or low tumor RSK2 expression is different for panels (A)/(B) versus (C). No conflicting results were found in other public glioma datasets. The graphs present patient groups separated at median RSK2 tumor mRNA expression. Y-axis presents survival probability, X-axis months of follow up. In all three datasets, high RSK2 expression is prognostic for poor outcome. This prognostic significance was also found for average RSK2 expression cut-off.

In this study, we performed *in vitro* experiments on both GBM cell lines and fresh GBM patient-derived cells, that all showed a role for RSK2 in GBM migration/invasion and therapy resistance. To verify that our results also apply *in vivo*, in human patients, we performed RSK2 mRNA expression analyses in large human glioma datasets in the public domain. We note that RSK2 mRNA studies can provide only surrogate markers for RSK2 enzymatic activity, but in the absence of large public datasets on enzymatic activity in cancer this is the best information available. First, by comparison of the brain samples in the large “Human Body Map” with the three largest, best annotated public glioma datasets, we observed that RSK2 mRNA expression is significantly lower in normal brain tissue than in human glioma samples (Figure 7), in concordance with a tumor-promoting role for RSK2 in glioma. Brain tissue is very heterogeneous, and RSK2 expression could vary considerably between the different brain tissue subtypes. However, we find that the variances in RSK2 expression in normal brain tissue and in the three human glioma tissue datasets are relatively small and comparable in size, suggesting that RSK2 expression variations also occur in glioma. Further support for higher expression of RSK2 in tumor tissue compared to normal tissue is obtained from comparing glioma tumor stages. We found that RSK2 tumor mRNA expression is higher in aggressive, metastatic, grade 4 tumors than in lower grade (2 and 3) tumors in three distinct patient cohorts (Figure 8). This also suggests that RSK2 expression in tumors is correlated to *in vivo* metastasis. Finally, using Kaplan-Meier analysis, we were able to determine that the survival of glioma patients with low tumor RSK2 expression is significantly higher than that of glioma patients that have high RSK2 expression in their tumors. This result was found for the three largest patient cohorts in the public domain (Figure 9), suggesting this correlation is robust. We conclude that *in vivo* high RSK2 expression is linked to metastasis and poor survival in complete agreement with our *in vitro* studies.

## DISCUSSION

Malignant gliomas are among the most aggressive and deadly forms of cancer and can affect any age group [3]. In high-stage glioma, GBM, infiltration of primary tumor cells into normal tissue and dissemination throughout the brain is a central but unmet challenge to successful treatment [38]. Indeed, patients with GBM respond poorly to the standard therapeutic regimen of radiotherapy and chemotherapy that follow tumor resection. The median survival is about 16 months [38]. It is therefore imperative to identify novel approaches to specifically attack GBM cell survival, proliferation and invasion. We have found that RSK2 can drive GBM migration and invasion. This appears to be mediated in part through RSK2 effects on FLNa and subsequent changes in integrin activity and cell adhesion. Inhibition of RSK isoforms in combination with temozolomide increased temozolomide effectiveness *in vitro*. As a first indication of the applicability of our *in vitro* results in new clinical routes, we find that RSK2 is up-regulated in human glioma cells, correlates with high-stage, metastatic disease, and is a significant predictor for poor patient survival. RSK inhibitors may therefore serve as new combination therapeutics.

We previously showed that RSK2 modulates cell adhesion and integrin activity and thereby regulates cell motility [14]. It does so in part by phosphorylating its target FLNa, a negative regulator of integrin activation [36, 47], and promoting FLNa association with the integrin cytoplasmic tails. We now find that RSK2 alters adhesion and migration in GBM cells. We find that RSK co-localizes with FLNa and that active RSK disturbs the initial adhesion of GBM cells to ECM. This likely contributes to the increased motility in these cells. RSK can also effect a migration program through transcription as well as changes in the actin cytoskeleton. We now find that these functions of RSK are active in GBM and that RSK inhibition limits GBM invasion.

RSK inhibitors generally affect all RSK isoforms (RSK1-4). However, not all RSKs control cell motility in the same way. While RSK2 often promotes migration of cancer cells [23], RSK1 has been shown to negatively regulate cell motility [48]. The observed effects in GBM cells might therefore be due to a broad inhibition of all RSK isoforms and their cellular functions. Thus, we tested the effect of individual RSK isoform gene knock-down in the GBM U373MG cell line and found that RSK2 can regulate invasion in these cells and in the temozolomide resistant primary patient-derived GBM cells. Additionally, the BI-D1870 inhibitor seems to block invasion more potently than gene knock-down of any specific RSK isoform. This may be due to a requirement for the activation of multiple RSK isoforms, redundancy in RSK isoform function, incomplete shRNA activity, or off-target effects of the inhibitor. Thus, additional studies are necessary to determine how each individual RSK isoform influences invasion and whether some of these isoforms have redundant functions in cell migration and invasion.

Further testing of RSK2 gene knock-down and the BI-D1870 inhibitor confirmed the ability to block migration in patient-derived GBM cell lines. Additionally, Kaplan-Meier analysis showed that high tumor RSK2 expression was prognostic for poor outcome, suggesting that RSK2 inhibitors like BI-D1870 may provide an interesting lead in targeting invasion in GBM treatment. The current standard of treatment is temozolomide combined with radiotherapy. We therefore tested whether combining RSK inhibition with temozolomide and radiotherapy had an additive effect. We found that RSK inhibition alone could reduce primary patient-derived GBM neurosphere survival and the combination of temozolomide with RSK inhibition was significantly more effective than either treatment alone. No additional effect of radiotherapy was noted for these cells. We note that our *in vitro* data and bio-informatics clinical data cannot substitute *in vivo* drug experiments, and that final confirmation of our findings awaits these studies. Currently there are no RSK inhibitors suitable for *in vivo* studies because their *in vivo* degradation kinetics are too unfavorable. Therefore, there is a pressing need to identify additional inhibitors that can maintain activity in animal models to do pre-clinical testing of RSK inhibitors.

Taken together, our study describes a novel function for RSK2 in the regulation of GBM cell motility and invasion. Therefore, inhibition of RSK2, and thereby metastasis reduction in GBM, is a promising new approach for addition to current standard therapies.

## MATERIALS AND METHODS

### Cell lines and reagents

Human astrocytes were purchased from ScienCell Research Laboratories (catalogue #1800, Carlsbad CA) and cultured according to manufacturer's instructions. Human glioblastoma (GBM) cell line U-87 MG was obtained from the NCI-Tumor Repository (Frederick, MD). U-373 MG was a gift from Dr. Santosh Kesari (UCSD School of Medicine, La Jolla, CA). Cells were cultured in D-MEM (U-87 MG, U-373 MG) or RPMI (SF-295; ATCC) (Mediatech, Manassas, VA). Basal media were supplemented with 10 % fetal bovine serum (HyClone, VWR, Radnor, PA), 1% non-essential amino acids and 1% penicillin/streptomycin (Cellgro, Manassas, VA). Cells were incubated in a humidified atmosphere containing 5% CO_2_ at 37°C. Transient transfections with the indicated constructs were performed using Lipofectamine (Life Technologies, Carlsbad, CA) according to the manufacturer's protocol. Antibodies specific for RSK2 (C-19), α-tubulin (DM1A); phospho p90RSK (Ser386) were from Life Technologies, antisera for RSK1 (#9333S), and RSK3 (#9343S) were from Cell Signaling; antiserum for RSK4 was from Abgent (AP7944a), and for FLNa (SAB4500951) from Sigma-Aldrich (St. Louis, MO). The RSK specific inhibitor SL0101 was purchased from Tocris Bioscience (Ellisville, MO), FMK was a gift from J. Taunton (University of California, San Francisco, CA) and BI-D1870 was obtained from BioVision Inc. (Milpitas, CA). Temozolomide (Temodar, S1237) was obtained from Selleckchem (Houston, TX).

### Isolation and culture of GBM primary cells derived from patients

Fresh human GBM material was acquired from GBM surgical patients and cultured as previously reported [45, 46]. Institutional Review Boards of the UC San Diego Human Research Protections Program reviewed and approved this project in accordance with the requirements of the Code of Federal Regulations on the Protection of Human Subjects. The IRB granted a waiver of informed consent for the recruitment component of this project. All analysis on human material and data was in compliance with the “Declaration of Helsinki for Medical Research involving Human Subjects” (www.wma.net/en/30publications/10policies/b3/index.html). Briefly, the dissociated tissue was washed, filtered through a 30 μm mesh and plated onto ultra-low adherence flasks at a concentration of 500,000 to 1,500,000 viable cells/ml. The stem cell isolation medium included human recombinant EGF (20 ng/ml), human bFGF (10 ng/ml) and heparin (2 μg/ml). Sphere cultures were then passaged by dissociation, washed, re-suspended in neural stem cell culture medium (#05750, Stemcell Technologies, Vancouver, Canada), and plated on ultra low-adherence 96-well plates at 2,000 cells per well for all subsequent drug testing. Alternatively, patient-derived dissociated GBM tissues were plated onto laminin-1 coated plates (Sigma, 3-5 μg/ml). Cell populations were dissociated using Acutase (Sigma) and expanded for 5-10 passages, then plated on regular flat bottom culture flasks.

### Lentiviral gene knock-down

U-373 MG cell lines with stable RSK isoform gene knock-down were generated using lentiviral particles containing shRNA expression constructs and subsequent selection with 4 μg/ml puromycin dihydrochloride (Santa Cruz Biotech). Lentiviral particles with pools of targeting sequences against RSK1 (sc-29475-V), RSK2 (sc-36441-V), RSK3 (sc-36443-V), RSK4 (sc-39212-V), or scrambled control shRNA lentiviral particles (sc-108080) were obtained from Santa Cruz Biotechnology and used according to the manufacturer's protocol. Lentiviral particles containing either pGIPZ non-silencing control or one of four shRNAs constructs targeting RSK2 (Thermo Scientific, Waltham, MA) were produced as previously described [49], and U-87 MG cells were transduced as previously described [50]. SK748 primary cells were transduced and selected with puromycin 1 μg/ml for 1 week.

### Transwell migration assay

The bottom side of cell culture inserts (8 μm pore size; Corning, Lowell, MA) was coated with 10 μg/ml human plasma fibronectin (Life Technologies). Cells were plated in the insert top chambers in serum free media, with the bottom chambers containing incomplete media supplemented with 10 ng/ml EGF. Cells were incubated for 18-20 hours at 37°C. The number of cells that migrated through the membrane was measured through Calcein-AM dye (Sigma-Aldrich) uptake and fluorescence at 495 nm excitation/515 nm emission. Transwell assays with primary GBM patient cells were performed by plating 10,000 cells in the top chambers of transwell inserts suspended in neural stem cell media supplemented with either DMSO or 5 μM BI-D1870, while the bottom chamber contained complete media supplemented with EGF and FGF. Cells were incubated for 24 hours at 37°C. The top chamber was fixed, stained with crystal violent and cell number was counted on both sides using an inverted microscope (Olympus).

### Cell migration scratch assay

Patient-derived GBM cells with RSK2 gene or control knock-down (shRSK2 or –NT) were grown in 6-well culture plates in neural stem cell media supplemented with either DMSO or 5 μM BI-D1870. The knockdown lentiviral vector expresses GFP to monitor transduced cells. The cells were allowed to grow to confluence then scratched with a 200 μL pipette tip. The cells were then washed in serum free media and replaced with media containing 1% serum. Cells were allowed to migrate into the scratch for various time points. Images of the scratch were acquired immediately after scratching (t=0) and at 24 hours. The average closure of the scratch by GFP-expressing migrating cells was measured using ImageJ software, and expressed as relative migration normalized to the t0 distance.

### Cell viability assay

Patient derived GBM8 cells with RSK2 gene or control knock-down (shRSK2 or –NT) were seeded on 24-well plates then irradiated at 0, 2 and 4 Gy the following day. After radiation, temozolomide was added to final concentrations of 1, 10, or 100 μM, and BI-D1870 to 1 or 10 μM. Cell viability was measured by Alarm Blue assay after 7 days of treatment. We also tested the combination of 1 μM temozolomide and 1 μM BI-D1870 on GBM8 control knock-down (ShNT) cells.

### Tumor spheroid invasion assay

Tumor spheroids were produced using U-87 MG cells as previously described [51] and embedded in 100% matrigel or a 50% matrigel/50% collagen mixture supplemented with either DMSO or 10 μM BI-D1870. Images were acquired 0, 24, and 48 hours after addition of the drug using an inverted microscope. The relative area of each spheroid was normalized (ImageJ) by dividing the area of a spheroid at 24 or 48 hours by its area at 0 hours.

### Adhesion assay

To measure cell adhesion, U-87 MG cells were harvested with a non-enzymatic cell dissociation buffer (Cellstripper, Cellgro, Mediatech) and plated in 96-well glass bottom plates coated with 10 μg/ml 3FN-(9–11) fusion protein. After the indicated amount of time pictures were taken using an IX81 microscope (Olympus) and an objective with 10x magnification. To quantify adhesion, the adherent cells in the pictures were counted.

### Immunofluorescence

Cells were grown on glass coverslips coated with 10 μg/ml fibronectin. At about 50 % confluency, cells were fixed in 2 % paraformaldehyde, permeabilized in 0.1 % Triton X-100 in PBS, and blocked with 1 % goat serum. Cells were incubated with primary antibodies for 1 hour. Alexa Fluor 488, 594 and 647 secondary antibodies (Life Technologies) were used to fluorescently label bound primary antibody. Coverslips were mounted on slides using Fluoromount G (Electron Microscopy Sciences, Hatfield, PA). Images were acquired on a Leica TCS-SP5 confocal microscope using a 62x oil-immersion objective (Leica Microsystems, Wetzlar, Germany).

### Immunoblotting

Cells were lysed with MLB lysis buffer (Millipore, Billerica, MA) as previously described [44]. Cell lysate protein was resolved by sodium dodecyl sulfate polyacrylamide gel electrophoresis (SDS-PAGE). Expression and phosphorylation of proteins was determined by immunoblotting using specific primary antibodies. Binding of primary antibodies was detected using IRDye 680 goat anti-mouse and IRDye 800 goat anti-rabbit secondary antibodies (Li-Cor Biosciences, Lincoln, NE). Bands were visualized using an Odyssey Infrared Imaging System (Li-Cor Biosciences). Scanned blots were cropped to improve clarity and conciseness of presentation using Adobe Photoshop CS (Adobe Systems Inc., San Jose, CA).

### Brain slice invasion assay

*Mus musculus* brain slices were cultured as previously described [52]. Whole brain slices of 300 μm thickness were placed on the membrane of six-well plate culture insert (PICM 030 5, Millipore) and washed once with PBS supplemented with magnesium (1 mM) and calcium (1 mM). The brain slices were cultured in 1 ml Eagle's MEM (111, Corning, Corning, NY), 25 mM HEPES, 25% HBSS, 25 % heat-inactivated horse serum, 6.5 mg/ml D-glucose, 100 U/ml penicillin, 100 μg/ml streptomycin, and 2.5 μg/ml amphotericin B). Thereafter, half of the medium was refreshed every three days. After the second change, the amount of culture medium was reduced to 800 μl. GBM cell lines (U373MG, U373MG (KO-RSK2), U373MG (NT-RSK2)) were labeled with the PKH67 fluorescent linker (MINI67, Sigma-Aldrich) according to the manufacturer's instructions. The tumor spheroids were formed by seeding 1 × 10^7^ labeled cells into a 60 mm ultra-low attachment dish (3261, Corning) containing 5 ml of D-MEM (10-013-CV, Corning) containing 10% FBS, 1X MEM nonessential amino acids (25025CL, Corning), 100 U/ml penicillin, 100 μg/ml streptomycin) and grown for several days. The day before implantation of the tumor spheroids, brain slices for inhibitor experiments were treated with 10 μM of RSK2 inhibitor BI-D1870 or vehicle. After 7 days, the viability of the brain slice was verified by the presence of the cortical lamination and hippocampal structure; then the insert was transferred to a well of a glass-bottom 6-well plate (P06G-1.5-20-F.S, MatTek, Ashland, MA). One small spheroid of approximately 200 μm was transferred to each brain slice as close to the corpus callosum as possible. The co-cultures were maintained for an additional 72 hours. The tumor cell invasion was followed using a confocal microscope (TCS SP5, Leica) and a stage-top incubator with digital gas mixer (TokaiHit WSKM/GM8000, Fujinomiya-shi Shizuoka-ken, Japan). The basal plane (0 μm) was determined 4 hours post implantation and serial sections every 20 μm downward from the basal plane to the bottom of the slice were imaged at the indicated time points. Propidium iodine staining of the brain slices at the end of the assay confirmed that the brain slice cultures had remained viable. To quantify the invasiveness of the spheroids, the density of the fluorescent signal was measured in each 20 μm section using ImageJ Software (National Institutes of Health, Bethesda, MD).

### Public human glioma mRNA expression dataset analysis

Human glioma mRNA expression datasets in the public domain were analyzed using R2: a genomics analysis and visualization platform developed in the Department of Oncogenomics at the Academic Medical Center – University of Amsterdam (http://r2.amc.nl). The human adult glioma datasets analyzed for this study; French-284 (GSE16011, [53]), Kawaguchi (GSE43378, unpublished), Sun-153 (GSE4290, [54]), and TCGA-540 (TARGET, [55]) were the only public datasets with WHO grade annotation and/or survival data. Human normal brain tissue samples were retrieved from the Roth-504 dataset (GSE7307, unpublished). Expression data (CEL files) were downloaded from the Gene Expression Omnibus (GEO) [56] on the NCBI website (http://www.ncbi.nlm.nih.gov/geo/) or from TARGET (https://ocg.cancer.gov/programs/target). Data were analyzed as described in [57]. Briefly, gene transcript levels were determined from data image files using GeneChip operating software (MAS5.0 and GCOS1.0, Affymetrix, Santa Clara, CA). Samples were scaled by setting the average intensity of the middle 96 % of all probe-set signals to a fixed value of 100 for every sample in the dataset, allowing comparisons between micro-arrays. The TranscriptView genomic analysis and visualization tool within R2 was used to check if probe-sets uniquely mapped to an anti-sense position in an exon of the gene (http://r2.amc.nl > genome browser). The probe-sets selected for RSK2 (203843_at for Affymetrix U133A and –P2, and 226335_at for Affymetrix U133P2) meet these criteria. For each dataset, the results obtained with the probe-set with the highest average expression in the highest amount of samples are shown, but the other probe-set never showed conflicting results. In addition, the independent Betastasis genomics analysis and visualization platform was queried for the REMBRANDT-523 human adult glioma Affymetrix U133P2 dataset (http://betastasis.com/glioma), using website standard settings. All expression values and other details for the datasets used can be obtained thru their identifiers from the NCBI GEO, TARGET, or Betastasis websites. Differential RSK2 mRNA expression between normal human brain and glioma tissue (Figure 7) was determined using a two-sided Student's t-test. RSK2 mRNA expression correlation with glioma WHO grade (Figure 8) was determined using the non-parametric Kruskal-Wallis test. The correlation between RSK2 tumor mRNA expression and survival probability (Figure 9) was evaluated by Kaplan-Meier analysis using the log-rank test as described [58]. For all tests, *P* < 0.05 was considered statistically significant.
